# Kinetics of α‑dicarbonyl compounds formation in glucose‐glutamic acid model of Maillard reaction

**DOI:** 10.1002/fsn3.1995

**Published:** 2020-11-08

**Authors:** Lili Zhang, Ying Sun, Dandan Pu, Yuyu Zhang, Baoguo Sun, Zhiyao Zhao

**Affiliations:** ^1^ College of Food Science and Engineering Tianjin University of Science and Technology Tianjin China; ^2^ Beijing Key Laboratory of Flavor Chemistry Beijing Technology and Business University Beijing China; ^3^ School of Artificial Intelligence Beijing Technology and Business University Beijing China

**Keywords:** 5‐hydroxymethylfurfural, glucose, glutamic acid, kinetics, α‐dicarbonyl compounds

## Abstract

As a potential health hazard, α‐dicarbonyl compounds have been detected in the thermally processed foods. In order to investigate the formation kinetics of α‐dicarbonyl compounds, liquid chromatography‐electrospray tandem mass spectrometry was employed to determine the content of α‐dicarbonyl compounds in glucose‐only and glucose‐glutamic acid (glucose‐Glu) thermal reaction models. The 3‐deoxyglucosone content was significantly higher than 6 α‐dicarbonyl compounds at 90–110℃, 0–6 hr in the two tested systems. The glutamic acid promoted the content accumulation of 1‐deoxyglucosone, diacetyl, methylglyoxal, and glyoxal, whereas inhibited the content of 3‐deoxyglucosone and 3,4‐dideoxyglucosone. Three‐fifths of the tested compounds content increased linearly with time increasing, but in glucose‐only system, the 1‐deoxyglucosone content increased logarithmically at 95–110℃ over reaction time. The formation of glucose (100–110℃, glucose‐only and glucose‐Glu), 5‐hydroxymethylfurfural (100–110℃, glucose‐only), 1‐deoxyglucose (105–110℃, glucose‐Glu), 3,4‐dideoxyglucosone (110℃, glucose‐Glu), glyoxal (95–110℃, glucose‐Glu) and diacetyl (90–95℃, glucose‐Glu) could be well fitted by exponential equation. Shortening the heating time and reducing heating temperature (except glyoxal in glucose‐only system) were the effective methods to decrease α‐dicarbonyl compounds content in the two tested systems. Additionally, high temperature could also reduce α‐dicarbonyl compounds content, such as 3‐deoxyglucosone (≥110℃, glucose‐only), 1‐deoxyglucosone (≥110℃, glucose‐only), glucosone (≥110℃, glucose‐only; ≥100℃, glucose‐Glu), methyloxyl (≥110℃, glucose‐only; ≥100℃, glucose‐Glu), diacetyl (≥110℃, glucose‐only), and glyoxal (≥100℃, glucose‐Glu).

## Graphical abstract

Kinetics of 7 α‐dicarbonyl compounds formation in glucose‐only and glucose‐glutamic acid systems was investigated. The formation kinetics of each compound varies with the heating temperature. Shortening the heating time and reducing heating temperature (except glyoxal in glucose‐only system) are effective method to reduce the content of α‐dicarbonyl compounds in the two systems tested

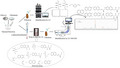

## 1 INTRODUCTION

α‐Dicarbonyl compounds are a class of highly reactive carbonyl compounds (Hamzalıoğlu & Gökmen, 2016). They are key precursors of food flavor and color substances. α‐Dicarbonyl compounds are important precursors of advanced glycation end products (AGEs), which are harmful to human body (Bulei et al., 2018; Daglia et al., 2013; El‐Maghrabey et al., 2018; Navarro et al., 2016). In addition, α‐dicarbonyl compounds are also involved in the formation of other toxic compounds (such as 5‐hydroxymethylfurfural (HMF), acrylamide, and 4(5)‐methylimidazole) during food processing, which significantly reduce the nutritional value of food (Jang et al., 2013; Knol et al., 2010; Lo et al., 2008; Yuan et al., 2008).

Meat flavorings based on the animal and vegetable protein as precursors are highly welcomed by domestic and foreign consumers. In the first half of 2020, the total operating revenue of 17 major domestic savory flavoring and seasoning companies reached 42.015 billion yuan (the data are from http://news.foodmate.net/2020/09/571161.html), which fully demonstrated the meat flavorings with various flavors dominate a huge market shares. Multiple carbon and nitrogen sources have been added during the Maillard reaction stage to obtain meat flavoring with good sensory quality. However, these preparation techniques involve many factors, including the reaction under high temperature, and adding acidic amino acid to the ingredient, which may promote the formation of α‐dicarbonyl compounds and HMF (Marianou et al., 2018; Zhang et al., 2019). Although researchers tried to control the production of α‐dicarbonyl compounds by adding polyphenoic extract without affecting the sensory properties of food (Pedreschi et al., 2018; Pu et al., 2020). In view of the negative effect of α‐dicarbonyl compounds and HMF on human health, many Maillard models have been established to obtain methods that can reduce α‐dicarbonyl compounds at the source. In the established model, glucose, fructose, maltose, lactose, and ribose are common sugars, and glycine, lysine, threonine, serine, and cysteine are common amino acids (Cha et al., 2019; Chen & Kitts, 2011; Jang et al., 2013). However, there are few models involving acidic amino acid especially glutamic acid. Glutamic acid is the most commonly used umami amino acid in the preparation of meat flavoring. Considering the promotion of acidic conditions on for α‐dicarbonyl compounds and HMF, it is important to monitor these compounds concentration under different thermal treatment conditions when making the meat flavoring.

To investigate the effect of acidic amino acid on the formation of α‐dicarbonyl compounds in meat flavoring processing, the sugar (glucose) and acidic amino acid (glutamic acid) commonly used in meat flavoring preparation were selected in this work. Unitary (glucose‐only) and binary (glucose‐glutamic acid) models were constructed. Due to the formation of α‐dicarbonyl compounds in a complex interconnected network, 10 main compounds (glucose, fructose, HMF, glucosone, 3‐deoxyglucosone, 1‐deoxyglucosone, 3,4‐dideoxyglucosone, glyoxal, methylglyoxal, and diacetyl) were observed. Liquid chromatography‐electrospray tandem mass spectrometry (LC‐ESI MS/MS) was employed to determine the α‐dicarbonyl compounds concentrations in all samples. This work could provide a promising strategy to reduce the amount of α‐dicarbonyl compounds produced by glucose‐glutamic acid system in meat flavoring.

## 2 MATERIALS AND METHODS

### 2.1 Materials and chemicals

Glucose (food grade) was provided by Jizhou Huayang Chemical Co., Ltd. Glutamic acid (food grade) was provided by Jizhou City Huaheng Biological Technology Co., Ltd. Formic acid (98%), glucose, and fructose standard were provided by Sinopharm Chemical Reagent Co., Ltd. High‐purity (99%) HMF standard was purchased from Sigma‐Aldrich. Ultra‐pure water was purchased from Hangzhou Wahaha Group Co., Ltd. Trifluoroacetic acid (TFA) (99.4%), methanol, and acetonitrile (HPLC grade) were all purchased from Thermo Fisher Scientific (Shanghai, China) Co., Ltd. Quinoxaline (98%) and 2‐methylquinoxaline (98%) were purchased from Aladdin Biochemical Technology Co., Ltd. 2,3‐Dimethylquinoxaline (98%) was purchased from TCI Development Co., Ltd. Diethylenetriaminepentaacetic acid (DETAPAC) (99%) and o‐phenylenediamine (99.5%) were purchased from Bailingwei Technology Co., Ltd. 2‐(2,3,4‐Trihydroxybutyl)‐quinoxaline was synthesized by Ontores Biotechnologies Co., Ltd. The 0.22 μm Nylon syringe filter was purchased from Bonna‐Agela Technologies Inc. (Venusil).

### 2.2 Preparation of model systems

In 250 ml four‐neck flasks, equipped with a reflux cooler, the systems of glucose‐only (0.3 M) and glucose‐Glu (0.3 M each) were prepared using water, respectively. Based on the study of the preparation of meat flavoring at atmospheric pressure, the temperature/time combination of this work was determined. Under the condition of standard atmospheric pressure, glucose‐only and glucose‐Glu systems were heated at 90, 95, 100, 105, and 110℃ for 0, 1, 2, 3, 4, 5, and 6 hr, respectively. The heating was carried out in a silicone oil bath. After the end of the reaction, samples were immediately cooled and stored at 4℃ before analysis. Each temperature/time combination was carried out in triplicates. Additional experiments were conducted when needed to obtain reliable results.

### 2.3 Analysis of glucose and fructose

The reaction mixture samples were diluted twofold with acetonitrile, filtered by 0.22 μm Nylon syringe filter, then determined the content of glucose and fructose by high performance liquid chromatography (HPLC).

The Agilent 1260 HPLC system (Agilent Corp.) equipped with a chromatography column (Innoval NH_2_, 250 mm × 4.6 mm i.d., 5 μm particle size) from Bonna‐Agela Technologies Inc. (Tianjin, China) was employed. The mobile phase was acetonitrile‐water (80:20, *v*/*v*); flow rate was 1.0 ml/min; injection volume was 20 μL; column temperature was 30℃; and the temperature of refractive index detector was 40℃. Glucose and fructose contents were calculated by an external calibration curve prepared with a concentration range from 28 to 7, 000 µg/ml.

### 2.4 Analysis of HMF

The reaction mixture samples were filtered by a 0.22 μm Nylon syringe filter, then the content of HMF was determined by HPLC. Quantitative analysis of HMF was carried out according to a validated method (Zhang et al., 2019; Zhang et al., 2017). An Agilent 1260 HPLC system (Agilent Corp) equipped with a chromatography column (Venusil XBP‐C18, 250 mm × 4.6 mm i.d., 5 μm particle size) from Bonna‐Agela Technologies Inc. (Venusil) was employed. The mobile phase was methanol–water (5:95, *v*/*v*); flow rate was 1.0 ml/min; detector wavelength λ was 284 nm; injection volume was 20 μL; and column temperature was 30℃. HMF contents were calculated by an external calibration curve prepared with a concentration range from 0.007 to 58.00 μg/mL.

### 2.5 Analysis of α‐dicarbonyl compounds

Derivatization of α‐dicarbonyl compounds was carried out with o‐phenylenediamine according to the methods of Kocadağlı and Gökmen (2016a). The derivatization of a 0.5 ml reaction mixture was performed by adding 150 μl of 0.1 M phosphate buffer (pH 7) and 150 μl of 0.2% ο‐phenylenediamine in 10 mM DETAPAC. After mixing, the mixture was kept in the dark at indoor temperature for 2 hr before analysis.

The quinoxaline derivatives of glucosone, 3‐deoxyglucosone, 1‐deoxyglucosone, 3,4‐dideoxyglucosone, glyoxal, methylglyoxal, and diacetyl were determined by LC‐ESI MS/MS according to the method of Kocadağlı et al. with minor modifications (Kocadağlı, & Gökmen, 2016a). The Agilent 1260 series HPLC system coupled with Agilent 6470A triple‐quadrupole mass spectrometer was used. The chromatographic separation was performed on an XBP C18 column (150 mm × 4.6 mm i.d., 5 μm) using a gradient mixture of (A) 0.5% formic acid in water and (B) 0.5% formic acid in methanol as the mobile phase at a flow rate of 0.6 ml/min at 30°C. The gradient mixture started from 30% B and increased to 60% B at 10 min, and then it decreased to 30% B at 2 min, and the 30% B remained for 3 min. The chromatographic run was completed within 15 min. The injection volume was 10 μL. The electrospray source had the following settings: drying gas (N_2_) flow of 10 L/min at 350°C, nebulizer pressure of 40 psig, and capillary voltage of 3,500 V. Fragmentor voltage was set to 100 V. MS data were acquired in the positive mode, and α‐dicarbonyl compounds were identified by selected ion monitoring (SIM) mode. The SIM ions [M + H]^+^ of the quinoxaline derivatives were as follows: glucosone, 251; 1‐ or 3‐deoxyglucosone, 235; 3,4‐dideoxyglucosone, 217; dimethylglyoxal, 159; methylglyoxal, 145; and glyoxal, 131. Dwell time was set at 50 ms for each compound.

The SIM ions of the quinoxaline derivatives of α‐dicarbonyl compounds were used for quantitation. Extracted ion chromatograms of the quinoxaline derivatives of α‐dicarbonyl compounds standard were shown in the supplementary material (Figure S1). Total ion chromatogram and extracted ion chromatogram of the quinoxaline derivatives of α‐dicarbonyl compounds identified in heated glucose‐only and glucose‐Glu systems were given in (Figure S2). The contents of quinoxaline, 2‐methylquinoxaline, 2,3‐dimethylquinoxaline, and 2‐(2,3,4‐trihydroxybutyl)‐quinoxaline) were calculated by means of external calibration curves in the range from 3.2 × 10^–3^ to 2 µg/ml, 3.2 × 10^–3^ to 2 µg/ml, 6.4 × 10^–4^ to 2 µg/ml and 8.2 × 10^–3^ to 2.1 µg/ml, respectively. Similarly, the calibration curve of 2‐(2,3,4‐trihydroxybutyl)‐quinoxaline was used for semi‐quantitation of glucosone, 1‐deoxyglucosone, and 3,4‐dideoxyglucosone quinoxaline derivatives, because both have the same proton‐accepting groups (Kocadağlı, & Gökmen, 2016a). All working solutions were prepared with water.

### 2.6 Kinetics and statistical analysis

Kinetics analysis was conducted according to the method described by Zhang et al. (2019) and Chi et al. (2011). In a non‐enzymatic browning reaction, there is an initial induction period corresponding to the stage at which α‐dicarbonyl compounds forms. After this relatively quick induction period, the content of α‐dicarbonyl compounds increases linearly, exponentially, or logarithmically with time, as shown in Equation 1, 2 and 3, respectively:

(1) $$C_{t}=C_{0}+k_{0}\times t$$

(2) $$C_{t}=C_{0}\exp\;\left(k_{1}\times t\right)$$

(3) $$C_{t}=C_{0}+k_{2}\times \text{In}\;t$$

where *C*_0_ refers to the initial content of compounds (µg/ml); *t* is the time of heat treatment (*h*); *C*_t_ is the content of compound (µg/ml) at time *t*; *k*_0_, *k*_1_, and *k*_2_ are rate constants (h^‐1^).

The concentrations of reactants and products were expressed as µg/ml of reaction mixture. The experimental data were compared with the mathematical model, and the dynamic model was evaluated by correlation coefficients (*R* ^2^), accuracy factor (*Af*), bias factor (*Bf*), root mean square error (*RMSE*), and sum of squares of the differences of the natural logarithm of observed and predicted values (*SS*) (Kavousi et al., 2015; McClure et al., 1993). In general, a value of *Af*, *Bf* and *R* ^2^ closer to 1, together with a lower value for *SS* and *RMSE*, indicated a better‐fit model.

Statistical calculation was performed by the statistical package SPSS 17.0 (SPSS Inc) for one‐factor analysis of variance (one‐way ANOVA). Data were expressed as means ± standard deviations of triplicate determinations. The mean values were considered significantly different at *p* < .05.

## 3 RESULTS AND DISCUSSION

### 3.1 Glucose consumption and effect of glutamic acid

**Figure 1:**
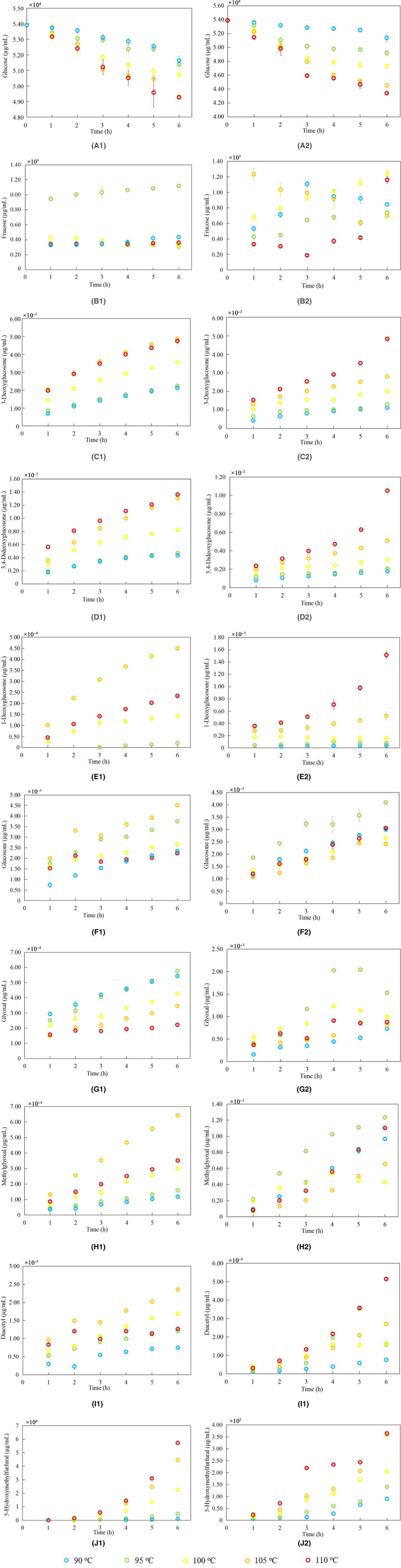
Changes in the content of 10 compounds in glucose‐only (1A–1J) and glucose‐Glu (2A–2J) model at different heating times and temperatures (Figure 1(2J) is from our previous work (Zhang et al., 2019))

The markers in Figure 1 respectively showed experimental observation data of reactants and products formed in heated glucose‐only and glucose‐Glu systems. Goodness‐of‐fit evaluation of glucose‐only and glucose‐Glu systems was shown in Table 1 and Table 2.

**Table 1:** Summary of kinetics analysis of 10 compounds formation in glucose‐only model

Compound	*T* ^a^ (℃)	Regression equation ^b^	*R* ^2^ ^c^	*Af* ^d^	*Bf* ^e^	*SS* ^f^	*RMSE* ^g^
Glucose	90	*C*(*t*) = −352.65*t* + 54,103 (0–6 hr)	0.9287	1.0000	1.0000	0.0001	211.0420
	95	*C*(*t*) = −368.05*t* + 53,874 (0–6 hr)	0.9397	1.0000	1.0000	0.0001	201.3949
	100	ln *C*(*t*) = −0.01*t* + ln53,693 (0–6 hr)	0.9718	1.0012	0.9988	0.0001	215.5312
	105	ln *C*(*t*) = −0.016*t* + ln54,122 (0–6 hr)	0.9531	1.0426	0.9592	0.0179	2,838.6362
	110	ln *C*(*t*) = −0.016*t* + ln53,920 (0–6 hr)	0.9891	1.0439	0.9580	0.0187	2,889.0131
Fructose	90	*C*(*t*) = 23.284*t* + 291.39 (1–6 hr)	0.9269	1.0002	0.9998	0.0057	12.2349
	95	*C*(*t*) = 32.75*t* + 926.16 (1–6 hr)	0.9743	1.0001	0.9999	0.0005	9.9448
	100	*C*(*t*) = −18.261*t* + 445.57 (1–6 hr)	0.9341	1.0002	0.9998	0.0030	9.0713
	105	*C*(*t*) = −7.3494*t* + 359.44 (1–6 hr)	0.9018	1.0001	0.9999	0.0010	4.5370
	110	*C*(*t*) = 3.9297*t* + 335.43 (1–6 hr)	0.7489	1.0001	0.9999	0.0007	4.2566
1‐Deoxyglucosone	90	‐					
	95	*C*(*t*) = 3 × 10^−5^ln(*t*) − 3 × 10^–5^ (3–6 hr)	0.9955	1.8226	0.5487	3.1453	0.0000
	100	*C*(*t*) = 7 × 10^−5^ln(*t*) + 3 × 10^–5^ (1–6 hr)	0.9847	1.0706	0.9340	0.0485	0.0000
	105	*C*(*t*) = 2 × 10^–4^ ln(*t*) + 1 × 10^–4^ (1–6 hr)	0.9986	1.0262	0.9745	0.0073	0.0000
	110	*C*(*t*) = 1 × 10^–4^ ln(*t*) + 4 × 10^–5^ (1–6 hr)	0.9859	0.0000	24,820.7909	3,518.2348	0.0000
3‐Deoxyglucosone	90	*C*(*t*) = 0.0028*t* + 0.0049 (1–6 hr)	0.9851	0.9953	1.0047	0.0203	0.0007
	95	*C*(*t*) = 0.0027*t* + 0.0063 (1–6 hr)	0.9936	0.9975	1.0025	0.0063	0.0004
	100	*C*(*t*) = 0.0041*t* + 0.0119 (1–6 hr)	0.9728	1.0033	0.9967	0.0222	0.0013
	105	*C*(*t*) = 0.0056*t* + 0.0173 (1–6 hr)	0.9690	1.0073	0.9928	0.0211	0.0019
	110	*C*(*t*) = 0.0054*t* + 0.017 (1–6 hr)	0.9670	1.0087	0.9914	0.0233	0.0019
3,4‐Dideoxyglucosone	90	*C*(*t*) = 0.0005*t* + 0.0016 (1–6 hr)	0.9197	0.9950	1.0050	0.0545	0.0003
	95	*C*(*t*) = 0.0006*t* + 0.0016 (1–6 hr)	0.9522	1.0451	0.9568	0.0384	0.0003
	100	*C*(*t*) = 0.0010*t* + 0.0028 (1–6 hr)	0.9234	1.0186	0.9818	0.0663	0.0005
	105	*C*(*t*) = 0.0018*t* + 0.0024 (1–6 hr)	0.9834	0.9955	1.0045	0.0356	0.0005
	110	*C*(*t*) = 0.0015*t* + 0.0047 (1–6 hr)	0.9809	0.9978	1.0022	0.0160	0.0004
Glucosone	90	*C*(*t*) = 0.0003*t* + 0.0005 (1–6 hr)	0.9847	0.9564	1.0455	0.0333	0.0001
	95	*C*(*t*) = 0.0004*t* + 0.0015 (1–6 hr)	0.9682	1.0205	0.9799	0.0143	0.0001
	100	*C*(*t*) = 0.0002*t* + 0.0015 (1–6 hr)	0.9804	1.0060	0.9941	0.0045	0.0001
	105	*C*(*t*) = 0.0005*t* + 0.0017 (3–6 hr)	0.9881	1.0448	0.9572	0.0086	0.0002
	110	*C*(*t*) = 0.0001*t* + 0.0014 (3–6 hr)	0.9473	0.9202	1.0867	0.0290	0.0002
Glyoxal	90	*C*(*t*) = 5 × 10^−5^ *t* + 0.0003 (1–6 hr)	0.9876	1.1105	0.9005	0.0738	0.0001
	95	*C*(*t*) = 6 × 10^−5^ *t* + 0.0002 (1–6 hr)	0.9931	0.9852	1.0150	0.0076	0.0000
	100	*C*(*t*) = 4 × 10^−5^ *t* + 0.0002 (1–6 hr)	0.9853	1.0831	0.9233	0.0441	0.0000
	105	*C*(*t*) = 4 × 10^−5^ *t* + 0.0001 (1–6 hr)	0.9880	0.9660	1.0352	0.0191	0.0000
	110	*C*(*t*) = 1 × 10^−5^ *t* + 0.0002 (1–6 hr)	0.9173	1.2423	0.8050	0.2924	0.0001
Methylglyoxal	90	*C*(*t*) =2 × 10^−5^ *t* + 1×10^–5^ (1–6 hr)	0.9870	1.0384	0.9630	0.0781	0.0000
	95	*C*(*t*) = 2 × 10^−5^ *t* + 2×10^–5^ (1–6 hr)	0.9960	0.9259	1.0800	0.0423	0.0000
	100	*C*(*t*) = 5 × 10^−5^ *t* + 2×10^–5^ (1–6 hr)	0.9914	1.0509	0.9515	0.0323	0.0000
	105	*C*(*t*) =1 × 10^−4^ *t* + 4×10^–5^ (1–6 hr)	0.9961	0.9779	1.0226	0.0129	0.0000
	110	*C*(*t*) =5 × 10^−5^ *t* + 4×10^–5^ (1–6 hr)	0.9977	0.1988	5.0314	16.9636	0.0002
Diacetyl	90	*C*(*t*) = 1 × 10^−6^ *t* + 1×10^–6^ (1–6 hr)	0.8671	0.8589	1.1643	0.4266	0.0000
	95	*C*(*t*) = 1 × 10^−6^ *t* + 4×10^–6^ (1–6 hr)	0.9733	0.8313	1.2030	0.2303	0.0000
	100	*C*(*t*) = 2 × 10^−6^ *t* + 4×10^–6^ (1–6 hr)	0.9880	0.9343	1.0704	0.0370	0.0000
	105	*C*(*t*) = 3 × 10^−6^ *t* + 8×10^–6^ (1–6 hr)	0.9496	1.1009	0.9084	0.0881	0.0000
	110	*C*(*t*) = 6 × 10^−6^ *t* + 9×10^–6^ (1–6 hr)	0.5097	0.8226	1.2156	0.3545	0.0000
5‐Hydroxymethylfurfural	90	‐					
	95	*C*(*t*)= 0.1183*t* − 0.2876 (2–6 hr)	0.9327	3.1641	/	/	0.0502
	100	ln *C*(*t*) = 0.8451*t* + ln0.0182 (2–6 hr)	0.9572	0.9988	1.0015	0.3194	0.3424
	105	ln *C*(*t*)= 0.8523*t* + ln0.0334 (2–6 hr)	0.9575	1.0011	0.9987	0.3223	0.5689
	110	ln *C*(*t*)=1.501*t* + ln0.0019 (1–6 hr)	0.8216	0.9846	1.0156	8.5636	4.3837

Note

‐: Multiple data were below the lowest detection limit of the analytical instruments, which makes it impossible for these models to be analyzed by the curve‐fitting method applied to the other model systems.

/: The data cannot be calculated by formula.

^a^ *T*: Heating temperature (℃).

^b^ *C*(*t*): the content of compound (µg/mL) at time *t*; t: time of heat treatment (h).

^c^ *R* ^2^: regression coefficient.

^d^ *Af*: accuracy factor.

^e^ *Bf*: bias factor.

^f^ *SS*: sum of squares of the differences of the natural logarithm of observed and predicted values.

^g^ *RMSE*: root mean square error. *R* ^2^, *Af*, *Bf*, *SS*, *RMSE*: indications of reliability and accuracy of models.

**Table 2:** Summary of kinetics analysis of 10 compounds formation in glucose‐Glu model

Compound	*T* ^a^ (℃)	Regression equation^b^	*R* ^2^ ^c^	*Af* ^d^	*Bf* ^e^	*SS* ^f^	*RMSE* ^g^
Glucose	90	*C*(*t*) = −364.74*t* + 53,960 (0–6 hr)	0.9162	1.0000	1.0000	0.0001	238.3391
	95	*C*(*t*) = −792.89*t* + 53,366 (0–6 hr)	0.8755	1.0001	0.9999	0.0010	645.8900
	100	ln *C*(*t*) = −0.023*t* + ln53,139 (0–6 hr)	0.9056	0.9997	1.0003	0.0015	783.8298
	105	ln *C*(*t*) = −0.034*t* + ln53,617 (0–6 hr)	0.9759	0.9995	1.0005	0.0008	534.6471
	110	ln *C*(*t*) = −0.036*t* + ln53,195 (0–6 hr)	0.9492	1.0014	0.9986	0.0020	862.7270
Fructose	90	*C*(*t*) = 57.971*t* + 641.37 (1–6 hr)	0.2915	1.0195	0.9809	0.2053	169.0825
	95	*C*(*t*) = 59.177*t* + 383.74 (1–6 hr)	0.7789	1.0053	0.9947	0.0504	58.9893
	100	*C*(*t*) = 110.91*t* + 576.35 (1–6 hr)	0.9956	1.0003	0.9997	0.0012	13.7565
	105	*C*(*t*) = −115.71*t* + 1,318.8 (1–6 hr)	0.8845	1.0038	0.9962	0.0593	78.2173
	110	*C*(*t*) = 133.13*t* − 4.1767 (1–6 hr)	0.5035	1.0152	0.9850	1.9560	247.3056
1‐Deoxyglucosone	90	*C*(*t*) = 3 × 10^−6^ *t* + 2×10^–5^ (2–6 hr)	0.9714	0.2618	0.9475	0.0263	0.0000
	95	*C*(*t*) = 9 × 10^−6^ *t* + 4×10^–5^ (1–6 hr)	0.5332	1.0705	0.9342	0.2141	0.0000
	100	*C*(*t*) = −5 × 10^−6^ *t* + 0.0002 (1–6 hr)	0.5319	1.1012	0.9081	0.0715	0.0000
	105	ln *C*(*t*) = 0.1323*t* + ln0.0002 (1–6 hr)	0.9697	0.8659	1.1548	0.1339	0.0001
	110	ln *C*(*t*) = 0.2910*t* + ln0.0002 (1–6 hr)	0.9701	0.8476	1.1798	0.2097	0.0002
3‐Deoxyglucosone	90	*C*(*t*) = 0.0013*t* + 0.0037 (1–6 hr)	0.9662	0.9910	1.0091	0.0297	0.0005
	95	*C*(*t*) = 0.0011*t* + 0.0061 (1–6 hr)	0.9199	1.0080	0.9921	0.0240	0.0006
	100	*C*(*t*) = 0.0018*t* + 0.0094 (1–6 hr)	0.9926	1.0071	0.9930	0.0181	0.0008
	105	*C*(*t*) = 0.0029*t* + 0.011 (1–6 hr)	0.9926	1.0040	0.9960	0.0041	0.0005
	110	*C*(*t*) = 0.006*t* + 0.008 (1–6 hr)	0.9437	0.9913	1.0088	0.0355	0.0028
3,4‐Dideoxyglucosone	90	*C*(*t*) = 0.0002*t* + 0.0007 (1–6 hr)	0.9919	1.0595	0.9438	0.0253	0.0001
	95	*C*(*t*) = 0.0002*t* + 0.001 (1–6 hr)	0.9581	1.0619	0.9417	0.0353	0.0002
	100	*C*(*t*) = 0.0003*t* + 0.0014 (1–6 hr)	0.9744	1.0343	0.9669	0.0161	0.0001
	105	*C*(*t*) = 0.0006*t* + 0.0014 (1–6 hr)	0.9942	0.9989	1.0011	0.0031	0.0001
	110	ln *C*(*t*) = 0.2785*t* + ln0.0017 (1–6 hr)	0.9694	0.9861	1.0141	0.0440	0.0007
Glucosone	90	*C*(*t*)= 0.0004*t* + 0.0009(1–6 hr)	0.9676	1.0418	0.9599	0.0371	0.0002
	95	*C*(*t*)= 0.0004*t* + 0.0016(1–6 hr)	0.9427	0.9824	1.0180	0.0265	0.0002
	100	*C*(*t*)= 0.0003*t* + 0.001(1–6 hr)	0.9945	1.0238	0.9767	0.0079	0.0001
	105	*C*(*t*)=0.0003*t* + 0.0007(1–6 hr)	0.9499	0.9813	1.0190	0.0248	0.0001
	110	*C*(*t*)=0.0004*t* + 0.0008(1–6 hr)	0.987	1.0351	0.9661	0.0177	0.0001
Glyoxal	90	*C*(*t*) = 0.0001*t* + 6×10^–5^ (1–6 hr)	0.9539	0.9793	1.0211	0.0549	0.0000
	95	ln *C*(*t*) =0.569*t* + ln0.0002 (1–4 hr)	0.9927	0.9709	1.0300	0.0154	0.0001
	100	ln *C*(*t*) = 0.2617*t* + ln0.0004 (1–4 hr)	0.9728	0.9634	1.0380	0.0152	0.0001
	105	ln *C*(*t*) = 0.1225*t* + ln0.0003 (1–4 hr)	0.9109	0.8785	1.1383	0.0745	0.0001
	110	ln *C*(*t*) = 0.1671*t* + ln0.0004 (1–6 hr)	0.7474	1.0952	0.9131	0.2148	0.0001
Methylglyoxal	90	*C*(*t*) = 0.0002*t* − 1 × 10^–4^ (1–6 hr)	0.998	1.1409	0.8765	0.1126	0.0001
	95	*C*(*t*) = 0.0002*t* + 1×10^–4^ (1–6 hr)	0.9479	1.0036	0.9964	0.1589	0.0001
	100	*C*(*t*) = 0.0001*t* + 1×10^–4^ (1–4 hr)	0.9725	0.9422	1.0613	0.0384	0.0000
	105	*C*(*t*) =0.0001*t* − 1 × 10^–4^ (1–6 hr)	0.9644	/	/	/	0.0001
	110	*C*(*t*) = 0.0002*t* − 2 × 10^–4^ (1–6 hr)	0.9698	/	/	/	0.0001
Diacetyl	90	ln *C*(*t*) = 0.3968*t* + ln(8 × 10^–6^) (1–6 hr)	0.9944	1.0584	0.9449	0.0349	0.0000
	95	ln *C*(*t*) =0.8168*t* + ln(6 × 10^–6^) (1–5 hr)	0.9897	0.9653	1.0359	0.0888	0.0000
	100	*C*(*t*) = 3 × 10^−5^ *t* + 2×10^–5^ (1–6 hr)	0.9046	1.1110	0.9001	0.1403	0.0000
	105	*C*(*t*) = 5 × 10^−5^ *t* − 4 × 10^–5^ (1–6 hr)	0.9755	0.9237	1.0826	0.8874	0.0000
	110	*C*(*t*) = 1 × 10^−4^ *t* − 1 × 10^–4^ (1–6 hr)	0.9425	/	/	/	0.0001
5‐Hydroxymethylfurfural ^h^	90	ln *C*(*t*) = 0.5722*t* + ln0.0030 (1–6 hr)	0.9701	1.1240	0.9869	0.1165	0.0061
	95	ln *C*(*t*) =0.5468*t* + ln0.0055 (1–6 hr)	0.9819	1.1796	0.9983	0.2227	0.0075
	100	ln *C*(*t*) =0.5304*t* + ln0.0113 (1–6 hr)	0.8996	1.2447	1.0035	0.3738	0.0348
	105	ln *C*(*t*) =0.5478*t* + ln0.0144 (1–6 hr)	0.9892	1.1227	0.9981	0.1475	0.0192
	110	*C*(*t*) = 0.0637*t* −0.0309 (1–6 hr)	0.9174	1.2194	1.0717	0.3297	0.0358

Note

/: The data cannot be calculated by formula.

^a^ *T*: Heating temperature (℃).

^b^ *C*(t): the content of compound (µg/ml) at time *t*; t: time of heat treatment (h).

^c^ *R* ^2^: regression coefficient.

^d^ *Af*: accuracy factor.

^e^ *Bf*: bias factor.

^f^ *SS*: sum of squares of the differences of the natural logarithm of observed and predicted values.

^g^ *RMSE*: root mean square error. *R* ^2^, *Af*, *Bf*, *SS*, *RMSE*: indications of reliability and accuracy of models.

^h^ The data of 5‐hydroxymethylfurfural are from our previous work (Zhang et al., 2019).

The initial contents of glucose in glucose‐only and glucose‐Glu systems were 53, 898.08 µg/ml and 53, 877.05 µg/ml, respectively (Figure 1 (1A and 2A)). In both the glucose‐only system and the glucose‐Glu system, the glucose content decreased with increasing heating time and increasing temperature. This is consistent with the trends that have been reported for glucose in glucose‐only and glucose‐NaCl systems (Kocadağlı, & Gökmen, 2016a). Levels of glucose decreased by 4.21% (90℃, 6 hr), 4.67% (95℃, 6 hr), 5.89% (100℃, 6 hr), 8.52% (105℃, 6 hr), and 8.59% (110℃, 6 hr) in glucose‐only system at five different heating temperatures, respectively. The glucose level in the glucose‐Glu system severally reduced by 4.66%, 8.66%, 12.21%, 17.35%, and 19.43%. The degradation rate of glucose content in the glucose‐Glu system was faster than that of glucose‐only system. Kocadağlı and Gökmen (2016a) also reported that the degradation of glucose was apparently faster in the presence of NaCl.

The kinetics analysis results of glucose consumption in the system of glucose‐only and glucose‐Glu were shown in Table 1 and 2, respectively. At the given temperature, the glucose consumption exhibited zero‐order kinetics at 90–95℃ (0–6 hr) in both glucose‐only and glucose‐Glu systems. And the consumption rate of glucose increased with the increase of temperature. In contrast, when the reaction temperature was higher than 100℃, the kinetics of glucose consumption was fitted by equation (2) in two systems. At the same temperature, the consumption rate of glucose in glucose‐Glu system was significantly higher than that of glucose‐only system. Therefore, it could be concluded that the presence of glutamic acid would accelerate the consumption of glucose.

### 3.2 Fructose formation and effect of glutamic acid

In aqueous solution system, glucose could convert to fructose and mannose by the intermediate of enediol. But the epimerization of glucose to mannose was less pronounced transformation than the aldose–ketose interconversion (Kocadağlı, & Gökmen, 2016b). Therefore, the fructose was the main product of glucose in glucose‐only and glucose‐Glu systems.

The average accumulation of fructose was about 478.15 µg/ml and 640.32 µg/ml in glucose‐only and glucose‐Glu systems after 1 hr of heating at five different heating temperatures, respectively (Figure 1 (1B and 2B)). Fructose was formed a very high initial rate, which was consistent with the previous literature (Kocadağlı, & Gökmen, 2016a). After 1 hr of heating, the highest content of fructose was 943.90 µg/ml (95℃, 1 hr) and 1, 232.95 µg/ml (105℃, 1 hr) in glucose‐only and glucose‐Glu systems, separately (Figure 1 (1B and 2B)). The formation content of fructose in the glucose‐only system was not obvious after heating for 1 hr, while the formation content of fructose in the glucose‐Glu system fluctuated wildly. The highest content of fructose was not observed at the highest temperature (110℃) in glucose‐only system, nor in glucose‐Glu system, which might be the accelerated degradation of fructose at high temperature.

The kinetics analysis results of fructose formation in the system of glucose‐only and glucose‐Glu were shown in Table 1 and 2, respectively. At the given temperature, the fructose formation exhibited zero‐order kinetics at 90–105℃ (1–6 hr) in glucose‐only system. The formation rate of fructose increased with the increase of temperature at lower heating temperature (90–95℃). When the heating temperature was 100 and 105℃, fructose consumption was greater than its formation. The content of fructose showed a trend of accumulation at 110℃. The formation of fructose could not be well fitted by zero‐order kinetics at 110℃ (1–6 hr) (*R* ^2^ = 0.7489). In the glucose‐Glu system, except for 100℃, the curve‐fitting of fructose formation was not ideal (*R* ^2^ < 0.9). The presence of acidic amino acids (glutamic acid) might contribute to the Maillard reaction, making the reaction of the entire system more complicated (Kavousi et al., 2015).

### 3.3 3‐Deoxyglucosone, 3,4‐dideoxyglucosone, 1‐deoxyglucosone, and glucosone formation and effect of glutamic acid

As shown in Figure 1 (1C–1F and 2C–2F), the contents of 4 C_6_ α‐dicarbonyl compounds increased with the increasing of heating time in glucose‐only and glucose‐Glu systems. In glucose‐only system, the highest contents of 3‐deoxyglucosone, 1‐deoxyglucosone and glucosone were observed with heating at 105℃, 6 hr (Figure 1 (1C, 1E and 1F)). The highest content of 3,4‐dideoxyglucosone was observed with heating at 110℃, 6 hr (0.0136 µg/ml), followed with heating at 105℃, 6 hr (0.0130 µg/ml) in glucose‐only system (Figure 1 (1D)). The highest contents of 3‐deoxyglucosone, 3,4‐dideoxyglucosone and 1‐deoxyglucosone were observed with heating at 110℃, 6 hr in the glucose‐Glu system (Figure 1 (2C, 2D and 2E)). The highest content of glucosone was observed with heating at 95℃, 6 hr in the glucose‐Glu system (Figure 1 (1F)). The content of 3‐deoxyglucosone, 1‐deoxyglucosone, and glucosone was decreased with heating at > 105℃ in glucose‐only system. The formation rate of 3,4‐dideoxyglucosone was also decreased with heating at > 105℃ in the glucose‐only system. In contrast with the glucose‐only system, the contents of 3‐deoxyglucosone, 3,4‐dideoxyglucosone, and 1‐deoxyglucosone were positively correlated with heating temperature in glucose‐Glu system. It was not conducive to the accumulation of glucosone when the heating temperature exceeded 95℃ in glucose‐Glu system.

3‐Deoxyglucosone, 3,4‐dideoxyglucosone, and glucosone were rapidly accumulated with the increasing of heating time at the beginning. The 3‐deoxyglucosone was predominant whether in glucose‐only system or in glucose‐Glu system at five different heating temperatures. The same observation was also reported in glucose‐lysine system (Gobert & Glomb, 2009). Gobert and Glomb (2009) also reported that 3‐deoxyglucosone‐quinoxaline increased independent from oxygen during the 7‐day observation period. The content of 3‐deoxyglucosone was the highest with heating at 121℃, 90 min in glucose‐glycine system, while at 121℃, 30 min in glucose‐lysine system (Chen & Kitts, 2011). The highest contents of 3‐deoxyglucosone in glucose‐only and glucose‐Glu systems were observed at the maximum heating time tested (Figure 1 (1C and 2C)), which was consistent with the study of 3‐deoxyglucosone in glucose–glycine system (Chen & Kitts, 2011). At five different heating temperatures, the highest content of 3,4‐dideoxyglucosone was 0.0136 µg/ml (110℃, 6 hr) in glucose‐only system (Figure 1 (1D)). The highest content of glucosone was 0.0045 µg/ml (105℃, 6 hr) in glucose‐only system (Figure 1 (1F)). However, the glucosone content was significantly higher than 3,4‐dideoxyglucosone at 90–95℃ in glucose‐Glu system. The 3,4‐dideoxyglucosone content was higher than the glucosone content with heating at > 100℃ in glucose‐Glu system. The highest content of 3‐deoxyglucosone (105℃, 6 hr) was about 108.36 times as high as that of 1‐deoxyglucosone (105℃, 6 hr) in glucose‐only system. The highest content of 3‐deoxyglucosone (110℃, 6 hr) was about 31.85 times as high as that of 1‐deoxyglucosone (110℃, 6 hr) in glucose‐Glu system. The highest content of glucosone was observed with heating at 105℃, 6 hr in glucose‐only system, while the highest content of glucosone was observed with heating at 95℃, 6 hr in glucose‐Glu system. The ratio of 3‐deoxyglucosone (105℃, 6 hr) to glucosone (105℃, 6 hr) was 10.80 in glucose‐only system. The ratio of 3‐deoxyglucosone (95℃, 6 hr) to glucosone (95℃, 6 hr) was 3.17 in glucose‐Glu system. It has been reported that the ratio of 3‐deoxyglucosone to 1‐deoxyglucosone was 3, the ratio of 3‐deoxyglucosone to glucosone was 20 during heating aqueous solution of glucose and alanine in pH 7 phosphate buffer under reflux. The ratio of 3‐deoxyglucosone to 1‐deoxyglucosone and the ratio of 3‐deoxyglucosone to glucosone had nothing to do with heating time (Hofmann, 1999). In this work, the ratio of 3‐deoxyglucosone to 1‐deoxyglucosone and the ratio of 3‐deoxyglucosone to glucosone were different from those reported by Hofmann (Hofmann, 1999). The difference might be due to the formation and degradation of α‐dicarbonyl compounds during heating had complex kinetics and thermodynamic behaviors under the experimental conditions in this work.

The kinetics analysis results of 4 C_6_ α‐dicarbonyl compounds formation in the system of glucose‐only and glucose‐Glu were shown in Table 1 and 2, respectively. At the given temperature, the kinetics of 3‐deoxyglucosone, 3,4‐dideoxyglucosone, and glucosone formation were fitted by equation (1) at 90–110℃, while the kinetics of 1‐deoxyglucosone formation was fitted by equation (3) at 95–105℃ in the glucose‐only system (Table 1). The fitting effect of equation (3) on the kinetics of 1‐deoxyglucosone formation was not ideal (*Af* = 0; *Bf* = 24,820.7909; *SS* = 3,518.2348) (Table 1). Similar to glucose‐only system, the formation kinetics of 3‐deoxyglucosone and gluconone was also fitted by equation (1) at 90–110℃ (Table 2). However, the formation kinetics of 3,4‐dideoxyglucosone and 1‐deoxyglucosone was only fitted by equation (1) at low heating temperatures (Table 2). At higher temperature, equation (2) had better fitting effect on the formation kinetics of 3,4‐dideoxyglucosone (110℃) and 1‐deoxyglucosone (105–110℃) (Table 2). The formation rate of 3‐deoxygluconone and 3,4‐dideoxygluconone increased with the increase of temperature in the glucose‐only system, especially when the heating temperature was lower than 105℃ (Table 1). At the 5 given temperatures, the promotion effect of high temperature on the formation of 1‐deoxyglucosone, 3‐deoxygluconone, and 3,4‐dideoxygluconone was significantly in glucose‐Glu system. The formation rate of glucosone was irregular with the change of heating temperature in both glucose‐only and glucose‐Glu systems, which might be related to the complexity of the Maillard reaction under the test condition. In the glucose‐only system, the promotion effect of high temperature on the formation of 1‐deoxyglucosone was significantly at 95–105℃, while the higher temperature reduced the formation rate of 1‐deoxyglucosone (Table 1).

### 3.4 Glyoxal, methylglyoxal, and diacetyl formation and effect of glutamic acid

The formation rates of shorter chain (C_2_, C_3_, and C_4_) α‐dicarbonyl compounds glyoxal, methylglyoxal, and diacetyl were different at five different heating temperatures in glucose‐only system (Figure 1 (1G–1I and 2G–2I)). The highest contents of methylglyoxal and diacetyl were observed with heating at 105℃, 6 hr, whereas the highest content of glyoxal was observed with heating at 95℃, 6 hr in glucose‐only system. When heating temperature was higher than 105℃, the formation rates of methylglyoxal and diacetyl were decreased in glucose‐only system. Martins et al. reported that the content of methylglyoxal increased by heating time (within 4 hr) at 100℃ in heated glucose/glycine equimolar solution (Martins & Van Boekel, 2005). The changing trend of methylglyoxal content (within 4 hr of heating) in the study of Martins et al. was consistent with that of methylglyoxal content in this study. Glyoxal and methylglyoxal, as intermediates of glycosylation and lipid peroxidation, were more likely to take part in secondary reactions and converted into advanced glycosylation products, advanced lipid peroxidation products, and secondary products at high temperatures (Andrewes, 2012). This might be the reason that the highest contents of glyoxal and methylglyoxal were not observed at the highest temperature in this study. From Figure 1 (1G), the generation of glyoxal was negatively correlated with heating temperature in glucose‐only system. The highest contents of glyoxal were observed with heating at 95℃, 5 hr in the glucose‐Glu system (Figure 1 (2G)). Glyoxal formation rate decreased with heating at 95℃, 5 hr in glucose‐Glu system. It was worth mentioning that the formation rate of diacetyl also decreased with heating at 95℃, 5 hr. The reasons for this result remained to be further studied. The contents of methylglyoxal were the highest observed with heating at 95℃, 6 hr in the glucose‐Glu system (Figure 1 (2H)).

For 3 shorter chain α‐dicarbonyl compounds, glyoxal was predominating at lower temperature (≤ 100℃) in glucose‐only system. The content of methylglyoxal exceeded glyoxal with heating at 105℃, 2 hr in glucose‐only system. The ratios of glyoxal to methylglyoxal were 4.59 (90℃, 6 hr), 3.62 (95℃, 6 hr), 1.42 (100℃, 6 hr), 0.54 (105℃, 6 hr), and 0.63 (110℃, 6 hr) in glucose‐only system at five different heating temperatures, respectively. Hofmann reported that the glyoxal concentration was the highest when heated for 10 min, after which other carbohydrate degradation products increased with the decrease of glyoxal in glucose and alanine aqueous solution (Hofmann, 1999). The contents of glyoxal, methylglyoxal, and diacetyl in glucose‐Glu system were higher than that of glucose‐only system. The contents of glyoxal in glucose‐Glu system were 1.33 (90 ℃, 6 hr), 2.65 (95℃, 6 hr), 2.33 (100℃, 6 hr), 2.32 (105℃, 6 hr), and 3.95 times (110℃, 6 hr) as high as that of glucose‐only system, respectively (Figure 1). The contents of methylglyoxal in glucose‐Glu system were 8.18 (90℃, 6 hr), 7.76 (95℃, 6 hr), 1.43 (100℃, 6 hr), 1.02 (105℃, 6 hr), and 3.14 times (110℃, 6 hr) as high as that of glucose‐only system, respectively. The highest content of diacetyl in glucose‐Glu system was about 21.81 times as high as that of glucose‐only system. Glyoxal was formed directly by oxidation of aldose or corresponding imine after reduction aldehyde. It was not derived from deoxyglycosones or from Amadori rearrangement products (Weenen, 1998).

The kinetics analysis results of 3 shorter chain α‐dicarbonyl compounds formation in the system of glucose‐only and glucose‐Glu were shown in Table 1 and 2, respectively. At the 5 given temperatures, the glyoxal, methylglyoxal, and diacetyl formation both exhibited zero‐order kinetics when heated for 1–6 hr in glucose‐only system. The promotion effect of high temperature on the formation of methylglyoxal and diacetyl was significantly at 95–105℃, while the higher temperature reduced the formation rate of methylglyoxal and diacetyl (Table 1). However, high temperature can inhibit the formation of glyoxal (95–110℃) in the glucose‐only system (Table 1). The methylglyoxal formation also exhibited zero‐order kinetics, but the promotion effect of high temperature on the formation of methylglyoxal was not significantly in the glucose‐Glu system (Table 2). The glyoxal formation was fitted to equation (1) at 90℃ (1–6 hr), while equation (2) at 95–105℃ (1–4 hr) in the glucose‐Glu system (Table 2). The fitting effect of the first‐order kinetics on glyoxal formation at 110℃ (1–6 hr) was not ideal (*R* ^2^ = 0.7474) (Table 2). The diacetyl formation was fitted to equation (2) at 90℃ (1–6 hr) and 95℃ (1–5 hr), while equation (2) at 100–110℃ (1–6 hr) in the glucose‐Glu system (Table 2). And the promotion effect of high temperature on the formation of diacetyl was significantly. From Tables 1 and 2, the formation rate of glyoxal, methylglyoxal, and diacetyl had a tendency to decline with the increase of temperature, except for diacetyl in glucose‐Glu system, possibly due to the high volatility of these compounds.

### 3.5 HMF formation and effect of glutamic acid

As one of the major degradation products of carbohydrates, the HMF was produced unexpectedly during thermal treatment of carbohydrate‐containing food (Zhang et al., 2019). Compared with glucose, the conversion of fructose to HMF was efficient, and fructose could synthesize HMF without catalyst. This might be caused by the energy barrier of HMF formed by isomerization of glucose to fructose and dehydration of the intermediate was lower than that of other pathways (Mayes et al., 2015). Fructose tended to form HMF via the fructofuranosyl cation pathway in the presence of glutamic acid. Based on our previous studies on the content of HMF in the glucose‐Glu system (Zhang et al., 2019), the formation of HMF in glucose‐only and glucose‐Glu systems was positively correlated with temperature and time. The highest content of HMF in glucose‐only system was 5.73 µg/ml (110℃, 6 hr) (Figure 1 (1J)). The content of HMF in glucose‐Glu system was much higher than that of glucose‐only system. The contents of HMF in glucose‐Glu system were 920.83 (90℃, 6 hr), 291.47 (95℃, 6 hr), 90.46 (100℃, 6 hr), 80.13 (105℃, 6 hr), and 63.56 times (110℃, 6 hr) as high as that of glucose‐only system, respectively. Based on situ NMR analysis, Li et al. have confirmed that the transformation of fructose to HMF was a highly selective reaction that proceeded through the cyclic fructofuranosyl intermediate pathway (Li et al., 2010).

The kinetics analysis results of HMF formation in the system of glucose‐only were shown in Table 1. In our previous studies, it can be known that the HMF formation exhibited first‐order kinetics at 90–105℃ (1–6 hr), while zero‐order kinetics at 110℃ (1–6 hr) in glucose‐Glu system (Zhang et al., 2019). In contrast with the glucose‐Glu system, the HMF formation exhibited zero‐order kinetics at 95℃ (2–6 hr), while first‐order kinetics at 100–105℃ (2–6 hr) and 110℃ (1–6 hr) in glucose‐only system. The promotion effect of high temperature on the formation of HMF was significantly at the five given temperatures. Although there were differences in the models of HMF fitted under different heating conditions, the formation of HMF increased with the increase of heating time and temperature.

In summary, based on the kinetics analysis, the content of three‐fifths of the tested compounds increased linearly with time. The content of 1‐deoxyglucosone increased logarithmically at 95–110℃ over reaction time in the glucose‐only system. The exponential equation had a good fitting effect on the formation of glucose (100–110℃, both glucose‐only and glucose‐Glu systems) and HMF (100–110℃, glucose‐only; 90–105℃, glucose‐Glu), 1‐deoxyglucose (105–110℃, glucose‐Glu), 3,4‐dideoxyglucosone (110℃, glucose‐Glu), glyoxal (95–110℃, glucose‐Glu), and diacetyl (90–95℃, glucose‐Glu).

## 4 CONCLUSION

The effect of glutamic acid on the formation of α‐dicarbonyl compounds in thermal reaction was investigated in this work. The content of 3‐deoxyglucosone was significantly higher than 6 α‐dicarbonyl compounds at 90–110℃, 0–6 hr in both glucose‐only system and glucose‐Glu system. The glutamic acid promoted the content accumulation of 1‐deoxyglucosone, diacetyl, methylglyoxal, and glyoxal, whereas inhibited the content of 3‐deoxyglucosone and 3,4‐dideoxyglucosone. The accumulation of 7 α‐dicarbonyl compounds was positively correlated with time in the two different tested systems. Shortening the heating time and reducing heating temperature (except glyoxal in glucose‐only system) were the effective methods to decrease the content of α‐dicarbonyl compounds in the two different tested systems. In addition, high temperature could also reduce the content of α‐dicarbonyl compounds, such as 3‐deoxyglucosone (≥110℃, glucose‐only), 1‐deoxyglucosone (≥110℃, glucose‐only), glucosone (≥110℃, glucose‐only; ≥100℃, glucose‐Glu), methyloxyl (≥110℃, glucose‐only; ≥100℃, glucose‐Glu), and diacetyl (≥110℃, glucose‐only). The results provide insight into the formation rate of α‐dicarbonyl compounds at various temperatures and provide guideline for reducing the content of α‐dicarbonyl compounds produced by the glucose‐Glu system in the preparation of meat flavoring.

## CONFLICTS OF INTEREST

The authors declare that there are no conflicts of interest.

## Supporting information

Supplementary Material
