# Supplementary material for: Kinetics of α‑dicarbonyl compounds formation in glucose‐glutamic acid model of Maillard reaction
Source: Food Sci Nutr. 2020 Nov 8;9(1):290–302. doi: 10.1002/fsn3.1995 (PMC7802556; doi:10.1002/fsn3.1995)

**Figure S1.** Extracted ion chromatograms of the quinoxaline derivatives of 2 μg/mL glyoxal (m/z 131), methylglyoxal (m/z 145) and diacetyl (m/z 159), and 2.1 μg/mL 3-deoxyglucosone (m/z 235).


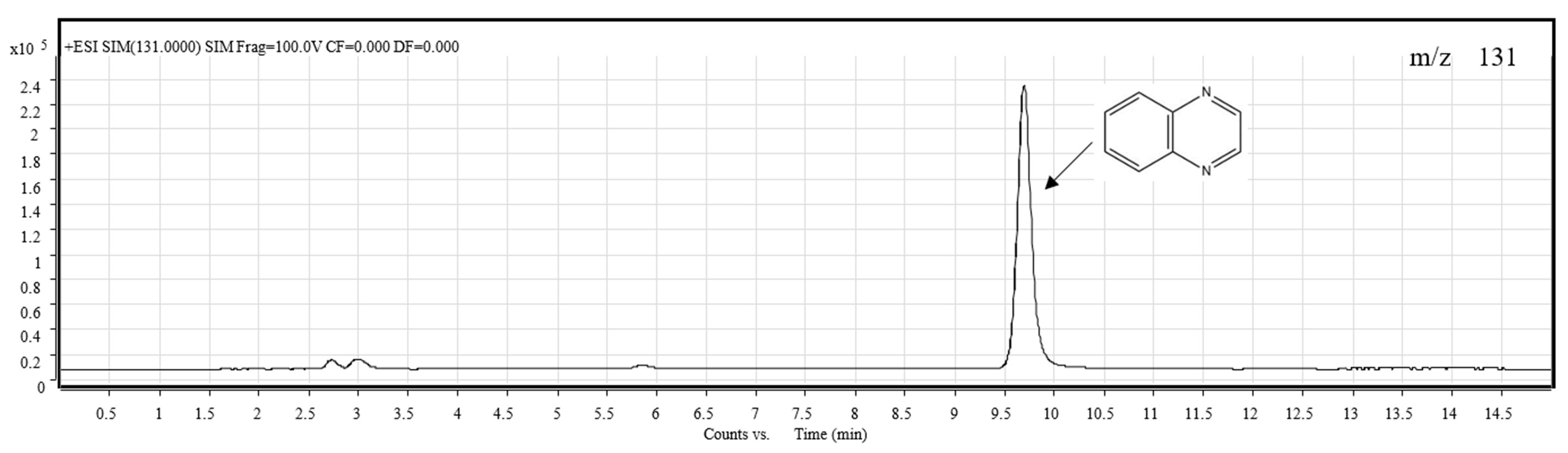


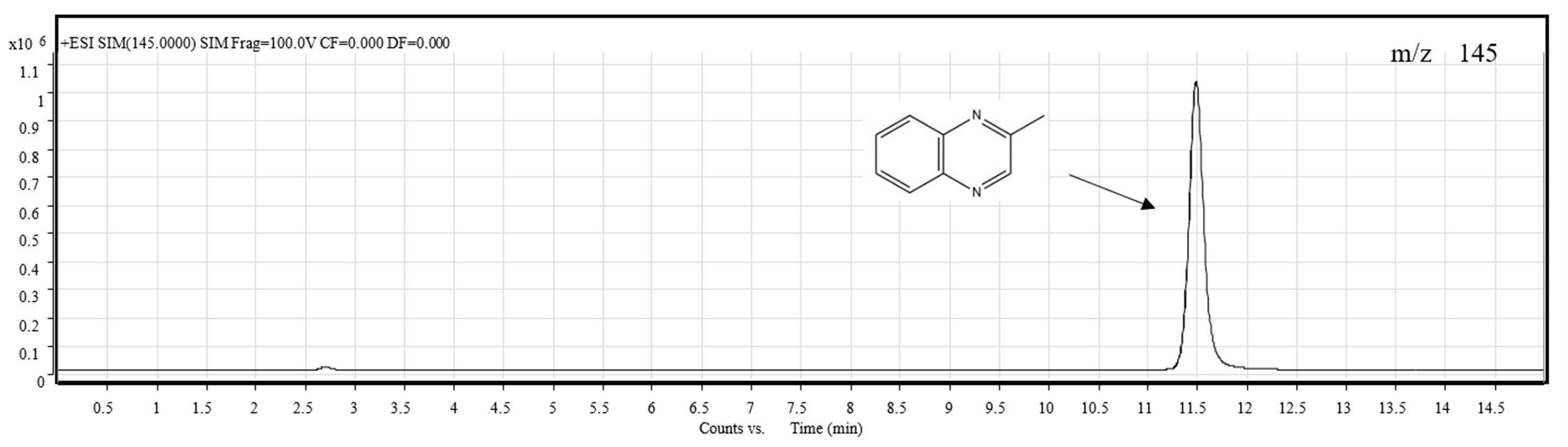

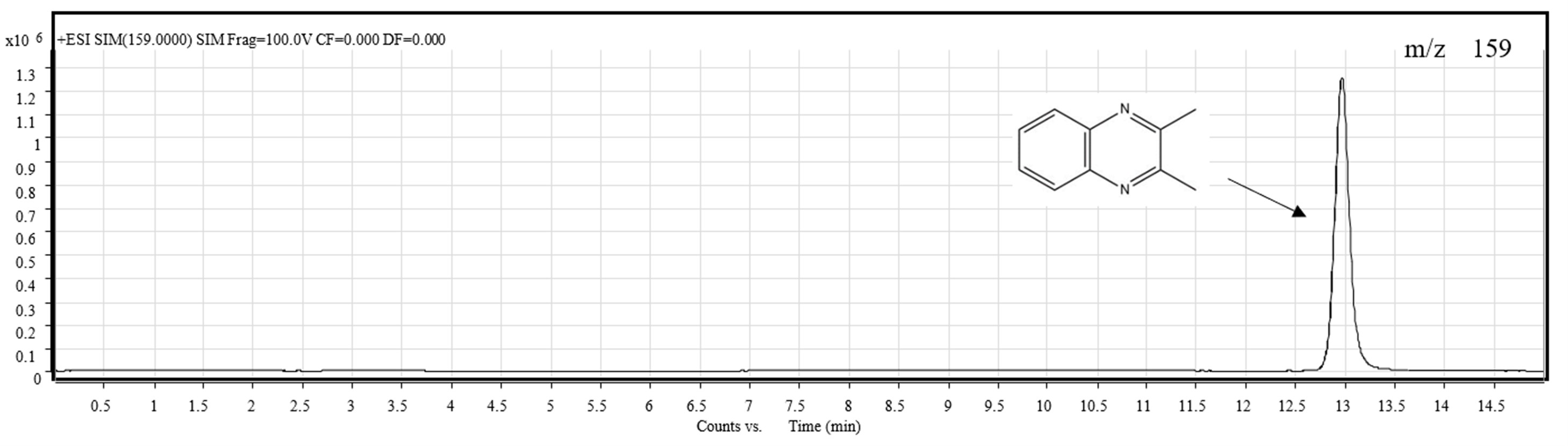

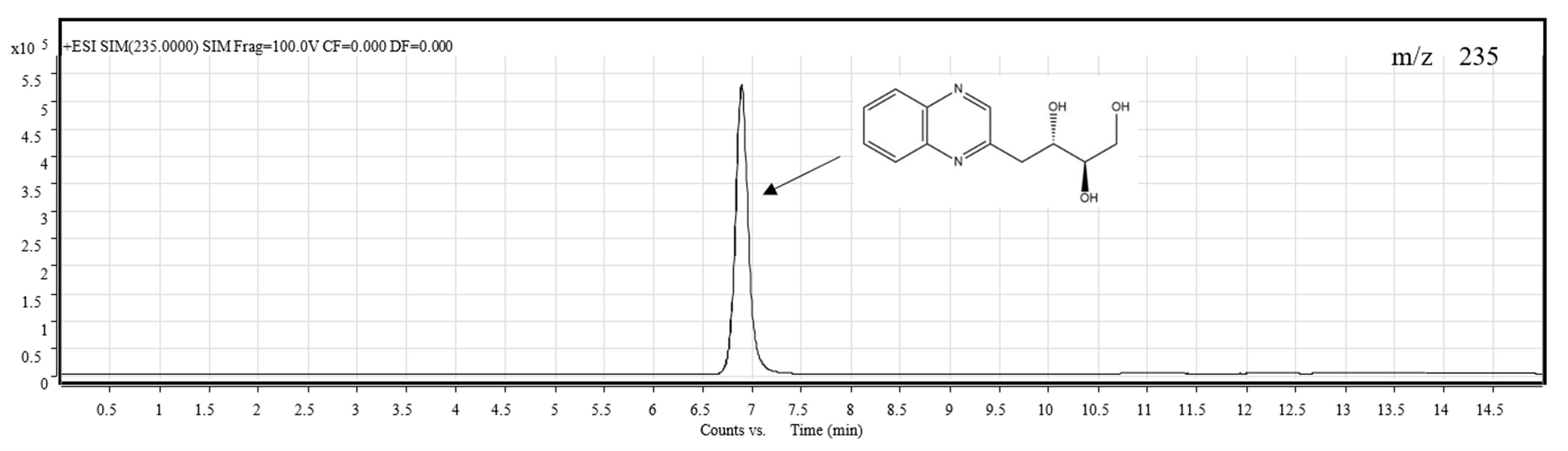


**Figure S2.** Total and extracted ion chromatograms of the quinoxaline derivatives of α-dicarbonyl compounds identified in a heated glucose and glucose-Glu mixture (90 ℃, 3 h). Glyoxal: m/z 131; methylglyoxal: m/z 145; diacetyl: m/z 159; 3,4-dideoxyglucosone: m/z 217; 1- or 3-deoxyglucosone: m/z 235; glucosone: m/z 251.


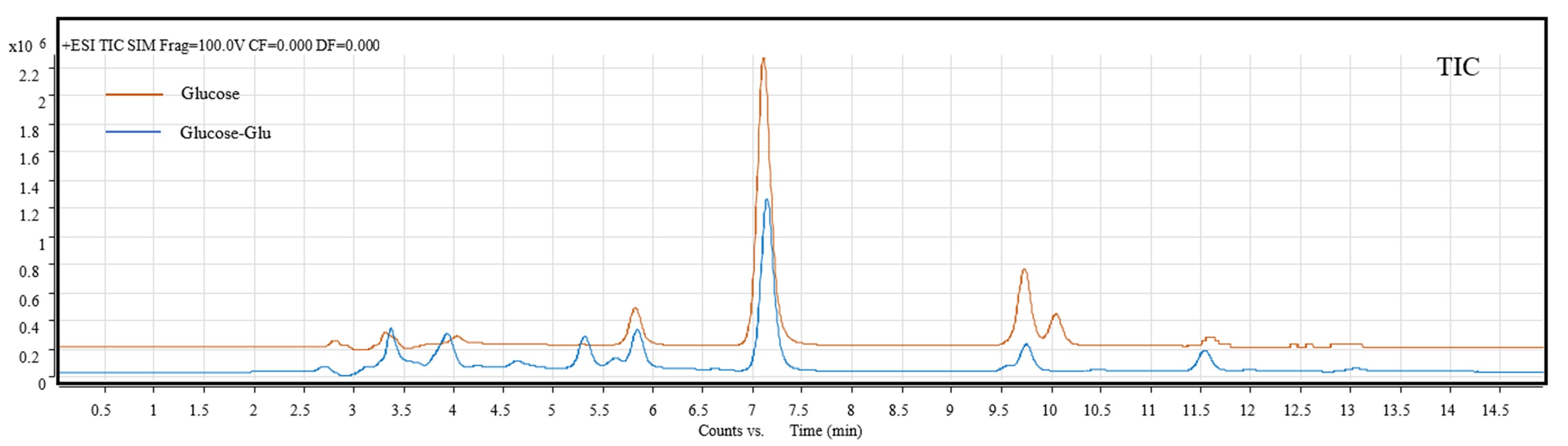

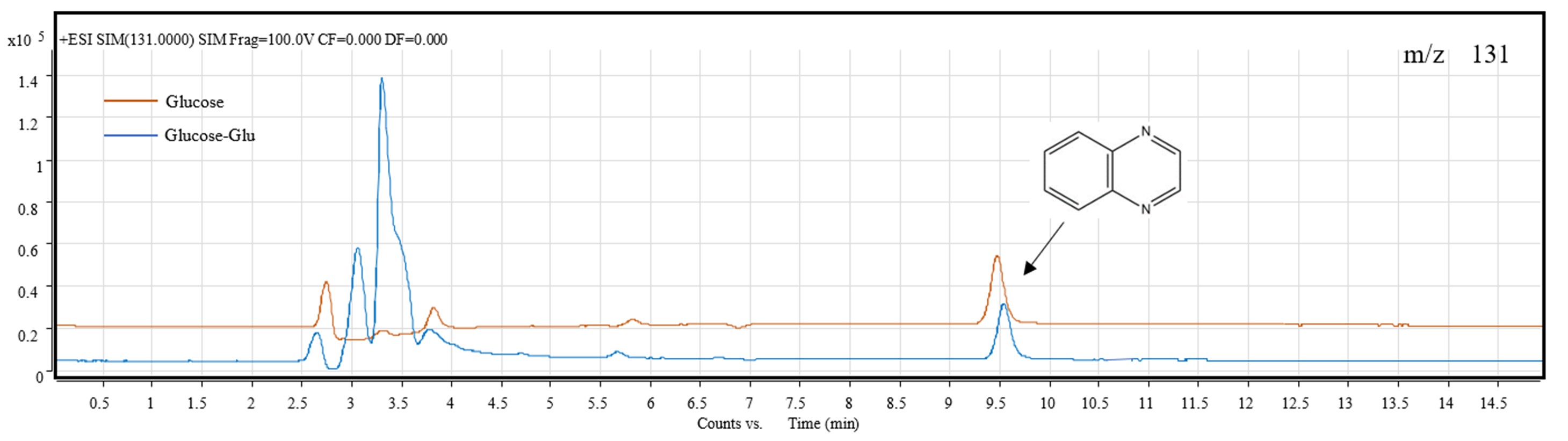

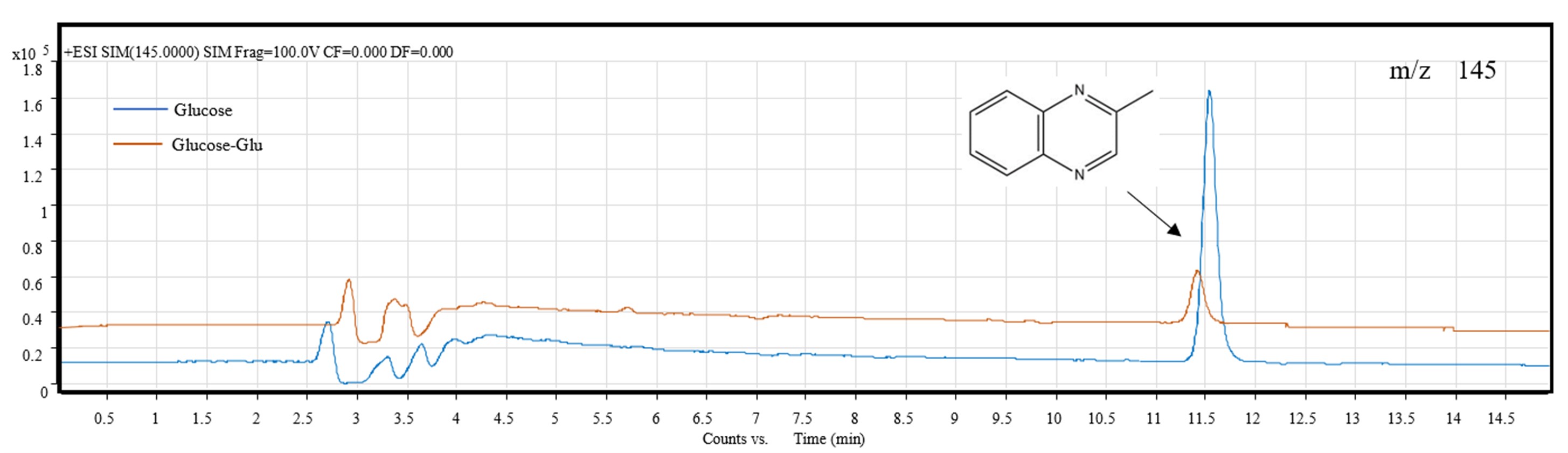

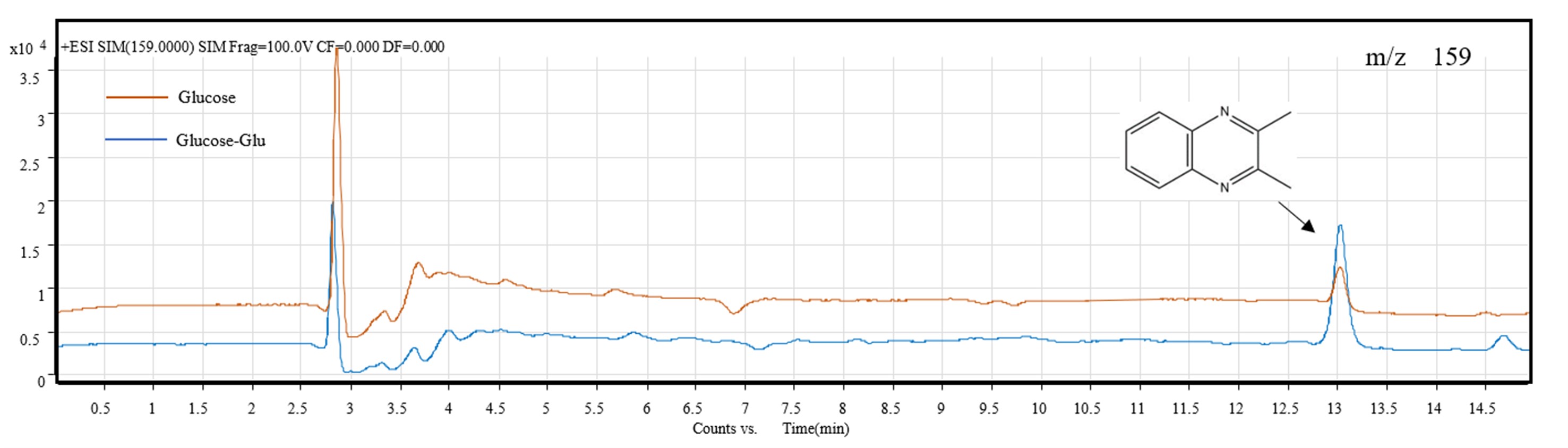

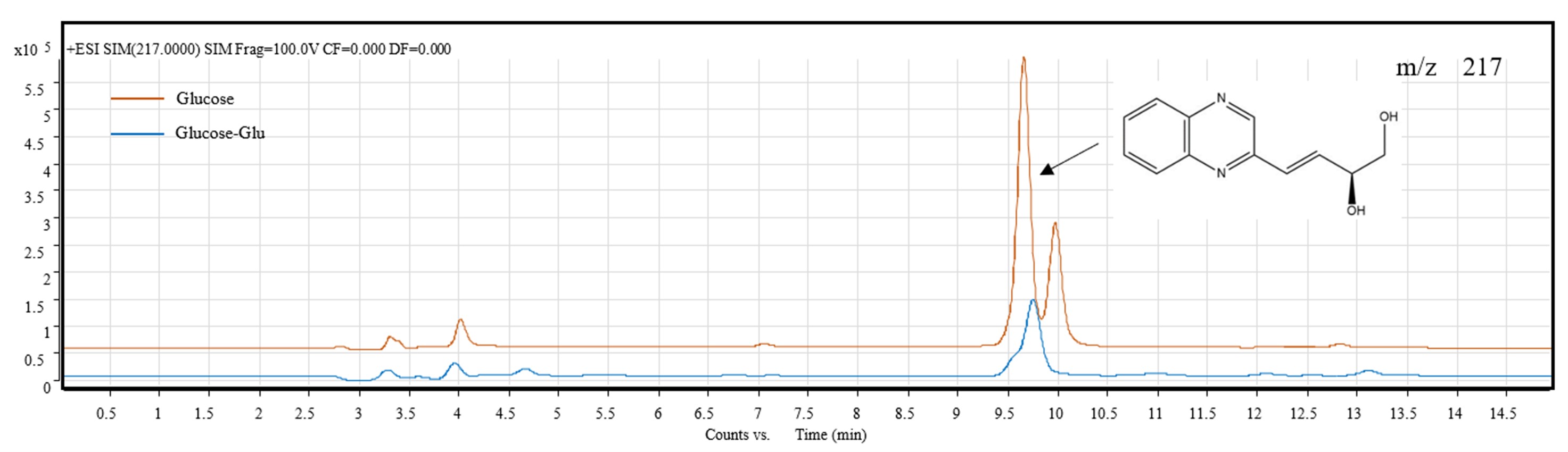

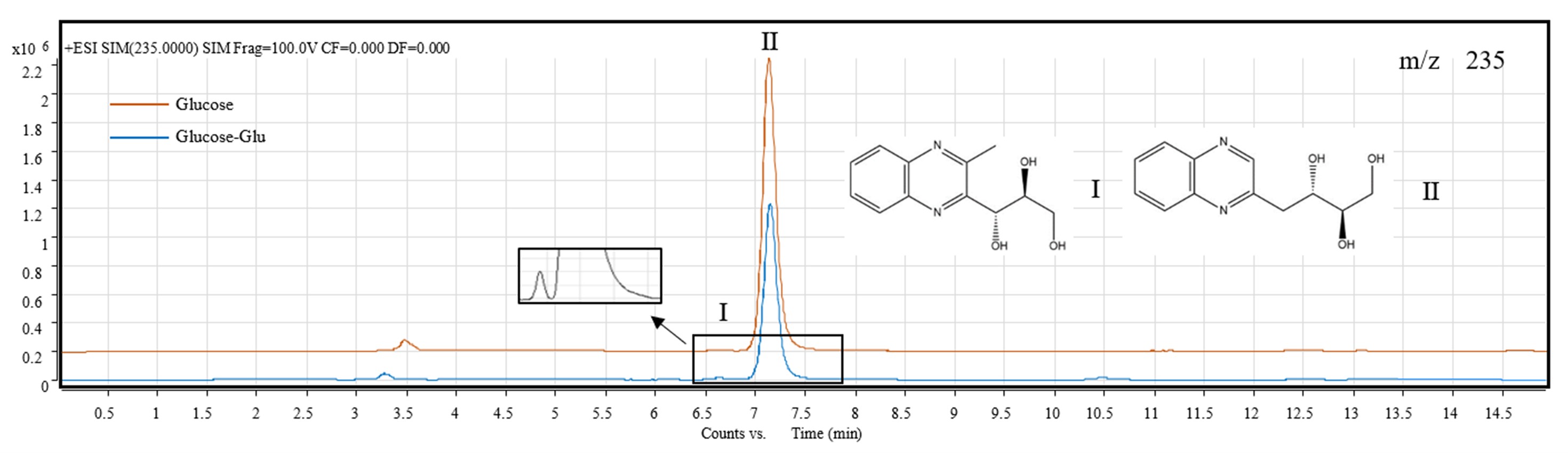

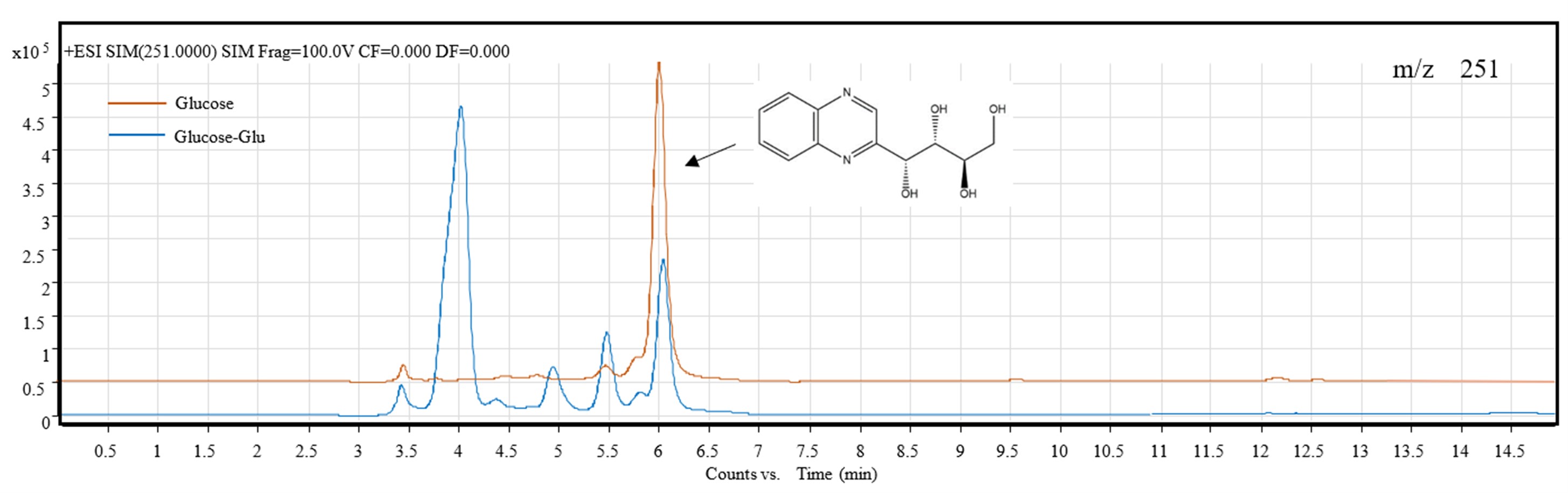

Supplement: Supplementary file 1 — Supplementary Material [file FSN3-9-290-s001.docx]
